# Supplementary material for: Early identification of recurrence in ovarian cancer: a comparison between the ovarian cancer metastasis index and CA-125 levels
Source: PeerJ. 2018 Nov 7;6:e5912. doi: 10.7717/peerj.5912 (PMC6228545; doi:10.7717/peerj.5912)
Supplement: File S1 [file peerj-06-5912-s001.docx]

Questionnaire on using the OCMI to identify the recurrence risk in ovarian cancer

**Question 1.** Are you willing to accept the free detection of OCMI?

If the answer is “yes”:

1. I hope to get further information about the disease, which helps to clarify my current situation and make me more rational.
2. I think it's trustworthy to use a scientific tool that contains computer programs.
3. I agree because it is free testing, but I will not care much about this index, because I have doubts about it. It is a new thing for me. I am worried about whether it is effective, and whether it can provide additional information for me and my doctor to make more rational decisions.

**Question 2.** If you agree to accept the free detection of OCMI, how long do you expect to get the test result?

1. Today
2. Two days or three days
3. A week
4. A month

**Question 3.** For the OCMI test results, you would like to inform:

A. only patient

B. only families

C. patient and families

**Question 4.** Are you willing to accept the disease management using OCMI?

1. yes
2. no

**Question 5.** Are you willing to follow up the ovarian cancer scientific research team and provide data only for scientific research? At the same time, the ovarian cancer scientific research team will provide you with free OCMI test and disease management?

A. yes

B. no
